# Motion estimation in PET-MRI based on dual registration: preliminary results for human data

**DOI:** 10.1186/2197-7364-1-S1-A39

**Published:** 2014-07-29

**Authors:** Michael Fieseler, Thomas Kösters, Christopher Glielmi, Fernando Boada, David Faul, Matthias Fenchel, Robert Grimm, Xiaoyi Jiang, Klaus P Schäfers

**Affiliations:** European Institute for Molecular Imaging, University of Münster, Münster, Germany; Department of Computer Science, University of Münster, Münster, Germany; Center for Advanced Imaging Innovation and Research, NYU Langone Medical Center, New York, USA; Siemens Medical Solutions USA, New York, USA; Siemens AG, Healthcare Sector, Kragujevac, Germany; Pattern Recognition Lab, University of Erlangen-Nürnberg, Erlangen, Germany

In current motion correction approaches in PET-MRI, motion information from PET data is neglected. We present an approach where PET and MRI data are used for motion estimation simultaneously. The presented approach has been evaluated on phantom data before [1]. Here, we present first results for human PET-MRI data.

The registration functional for dual registration is given by
1

Here, R_MR_ and R_PET_ denote two reference volumes and T_MR_ and T_PET_ the template volumes to be registered, D is a distance functional, and S is a regularizer. The scalar value β allows to weight the influence of the data term for PET [1]. The functional has been implemented using the FAIR toolbox [3].

Five patients were scanned following a clinical FDG scan. A self-gated radial VIBE sequence [2] and PET Listmode data were acquired. The datasets were re-binned into 5 coinciding PET and MRI phases (gates).

Registration were computed for β ∈ {0, 0.5, 1, 2}, α was chosen empirically as α = 20.

Correlation coefficients were computed for the heart region.

In Figure 2a we show correlation values for each gate of dataset 4. In all gates the correlation of the PET data is improved using the joint motion estimation approach using a weight of β = 2. In 2b average correlation values of all gates are shown for all datasets processed.

**Figure 1:**
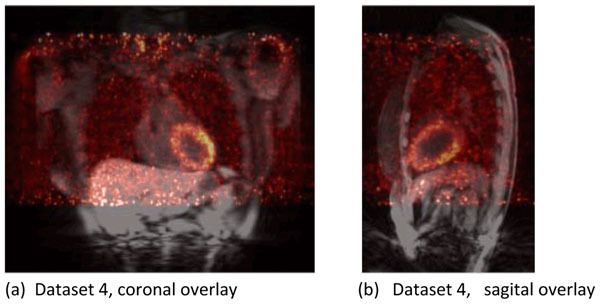
Overlay of PET and MR data for dataset 4, first respiratory phase (gate).

**Figure 2:**
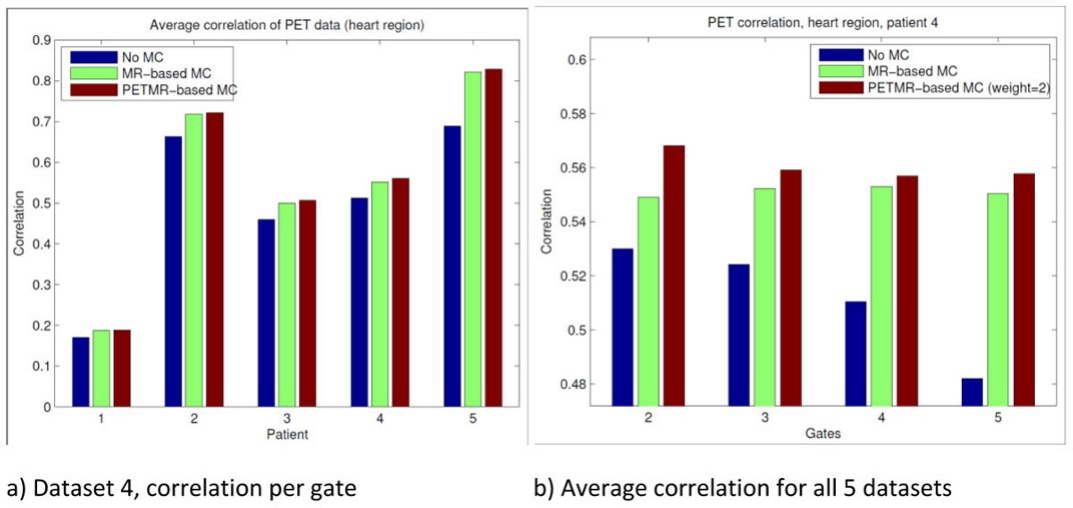
Correlation values for PET data. (a) Correlation values for the heart region in gates 2 to 5 for dataset number 4. (b) Average correlation values for all patients

We have shown that using a joint motion estimation approach the correlation of PET data is improved compared to an estimation of the motion solely on MRI data. Currently, we are evaluating motion-correcting reconstructions using the motion estimates from the proposed method.
